# Supplementary material for: Crosslinking Strategies for the Microfluidic Production of Microgels
Source: Molecules. 2021 Jun 20;26(12):3752. doi: 10.3390/molecules26123752 (PMC8234156; doi:10.3390/molecules26123752)
Supplement: Supplementary file 1 [file molecules-26-03752-s001.zip › molecules-1243250-supplementary.pdf]

molecules

MDPI

Supplementary Materials

Table S1. Microgels manufactured by internal gelation in microfluidic devices. ABIL EM 90: Silicone-based non-ionic W/O emulsifier from Evonik; AFFD: Axisymmetric flow-focusing device; BODIPY: a fluorescent dye; DMC: dimethyl carbonate; DMEM: Dulbecco's modified Eagle's medium; EDTA: Ethylenediaminetetraacetic acid; FA: ferulic acid; GDL: Glucono-1,5-lactone; MFFD: Microfluidic flow focusing device; Span 80: Sorbitan monooleate (Polysorbate 80); TAP: TRIS acetate phosphate; (d = diameter of beads, DP = dispersed phase, CP = continuous phase)

Microfluidic device	Microgels	W/O Emulsion formulation	Ref.
MFFD	Ca-alginate beads (d=60-110 µm)	DP: 2 wt% sodium alginate + 0.1 wt% $CaCO_3$ in water<br>CP: 5wt% CH_3COOH + 3 wt% Span 80 in soybean oil	[1]
Microfluidic Y-junction	Ba-alginate beads (d=249-318 µm)	DP: 2 % (w/v) sodium alginate + 5-10 mM $BaCO_3$ in water<br>CP: 0.15 vol% CH_3COOH in sunflower oil	[2]
T-junction	Ca-alginate beads (d=94-150 µm)	DP: 2 wt% sodium alginate + 1.14-2.27 mg/mL $CaCO_3$ in water<br>CP: 0.67-2.68 µL/mL CH_3COOH + 2 wt% lecithin in corn oil	[3]
MFFD	Ca-alginate beads (d=10-50 µm)	DP: 2 wt% sodium alginate + 50 M Ca-EDTA in water<br>CP: 0.05 vol% acetic acid + 1 wt% biocompatible surfactant in fluorinated carbon oil	[4]
Ψ-junction with wavy collection channel	Ca alginate beads loaded with fluorescent quantum dots (d=43-45 µm)	DP: Sodium alginate + $CaCO_3$ in water<br>CP: acetic acid + ABIL EM 90 in hexadecane	[5]
3D microfluidic AFFD	Ca alginate beads containing *Chlamydomonas* (d=82-108 µm)	DP: 1 wt% sodium alginate + 150 mM GDL + $CaCO_3$ + *Chlamydomonas* in TAP buffer<br>CP: 2 wt% lecithin in corn oil	[6]
MFFD	Ca pectin beads (d≈42 µm)	DP: 5-20 µg/µL Pectin + 2.5-10 µg/µL $CaCO_3$ in water<br>CP1: Anhydrous DMC or DMC saturated with water<br>CP2: 0.5 wt% acetic acid in DMC	[7]
MFFD with droplet trappers	Ca-alginate beads (d=100-200 µm)	DP: 1 wt% sodium alginate + 3 wt% $CaCO_3$ in blue dyed water<br>CP1: 1-2 wt% Span 80 in light mineral oil<br>CP2: 1 wt% acetic acid +1-2 wt% Span 80 in light mineral oil	[8]
Adhesive film-based microfluidic device	Ca-alginate beads (d≈90 µm)	DP: 1.5% (v/v) sodium alginate + 50 mM Ca-EDTA in water<br>CP1: 1% (v/v) Tween 80 in mineral oil<br>CP2: 1.4% (v/v) acetic acid + 1% (v/v) Tween 80 in mineral oil	[9]

Novel microfluidic flow-focusing shielded junction	Ca-alginate beads containing HEK293, U-2 OS and PC12 cells (d=75-100 μm)	DP: 2 wt% sodium alginate + 0. 5wt% $CaCO_3$ + 1×10^6 mL^{-1} cells in DMEM<br>CP1: 0.3 vol% acetic acid in sunflower oil<br>CP2: Pure sunflower oil	[10]
Upstream Y-junction combined with two downstream serial Ψ-junctions	FA-pectin/Bodipy-pectin Janus beads (d≈92 μm)	DP1: 2 wt% citrus FA-pectin +1 wt% $CaCO_3$ in water<br>DP2: 2 wt% citrus Bodipy-pectin +1 wt% $CaCO_3$ in water<br>CP: 0.5 wt% CH_3COOH+ 1 wt% Span 80 in sunflower oil	[11]
Upstream Y-junction combined with two downstream Ψ-junctions	FA-alginate/BODYPI-pectin Janus beads (d≈92 μm)	DP1: 2 wt% citrus BODYPI-pectin +1 wt% $CaCO_3$ in water<br>DP2: 2 wt% sodium FA-alginate +1 wt% $CaCO_3$ in water<br>CP: 0.5 wt% CH_3COOH + 1 wt% Span 80 in sunflower oil	[11]

Table S2. Microgels manufactured by external gelation in microfluidic devices. AFFD: axisymmetric flow focusing device; SY-Glyster CRS-75: Polyglycerol esters of fatty acids from Sakamoto Yakuhin Kogyo; DMC: dimethyl carbonate; DMEM: Dulbecco's modified Eagle's medium; HBSS: Hanks' balanced salt solution; α-MEM: Modification of Minimum Essential Medium that contains non-essential amino acids; MFFD: microfluidic flow focusing device; SAP: self-assembling peptide.

Microfluidic device	Microgels	Emulsion formulation	Ref.
MFFD	Ca-alginate beads (d=50-70 μm)	DP: 2 wt% sodium alginate in water<br>CP: 2 wt% $Ca(CH_3COO)_2$ + 3 wt% Span 80 in soybean oil	[1]
MFFD	Ca-alginate beads containing PS particles (d=30-230 μm)	DP: 1-2 wt% sodium alginate + 2×10^6 mL^{-1} PS particles in water<br>CP: 0.2-0.5 wt% CaJ_2 in undecanol	[12]
MFFD	κ-carrageenan beads (d≈50 μm)	DP: 0.8 wt% κ-carrageenan in water<br>CP: 0.25 wt% CaJ_2 in undecanol	[12]
MFFD	Carboxymethylcellulose beads (d≈80 μm)	DP: 1 wt% carboxymethylcellulose in water<br>CP: 0.25 wt% $Fe(NO_3)_3$ in undecanol	[12]
MFFD	Ca-pectate beads containing gold nanoparticles	DP: 0.33-0.66 wt% pectin + 5 nm gold nanoparticles in water<br>CP: 1-10 wt% acetic acid + 0.05-2 wt% $CaCO_3$ in rapeseed oil	[13]
Cross junction	Ca-pectate beads (d=65-78 μm)	DP: 10 wt% pectin in water<br>CP: 4M $CaCl_2$ emulsified and dehydrated + 2 wt% Span 80 in mineral oil	[14]
Cross junction	Ca-pectate beads containing Fe_2O_3 nanoparticles	DP: 5 wt% pectin + Fe_2O_3 nanoparticles in water<br>CP: 4M $CaCl_2$ emulsified and dehydrated + 2 wt% Span 80 in mineral oil	[14]

MFFD	Ca-pectate beads (d≈42 μm)	<u>DP</u>: 5, 10, 20 μg/μL pectin in water<br><u>CP</u>: DMC saturated with 9 μg/μL $CaCl_2$ aqueous solution<br><u>Gelation bath</u>: 20 μg/μL $CaCl_2$ in water	[7]
MFFD	Ca-alginate beads	<u>DP</u>: 2 wt% sodium alginate in water<br><u>CP</u>: $CaCl_2$ nanoparticles + 2 wt% Span 80 in mineral oil	[15]
MFFD with droplet trappers and two parallel oil flows	PuraMatrix™ peptide beads (d=100-200μm)	<u>DP</u>: 0.25-0.5 wt% PuraMatrix™ SAPs in water with blue food colour<br><u>CP1</u>: 1-2 wt% Span 80 in light mineral oil<br><u>CP2</u>: 1 g/mL Sudan dye + 1-2 wt% Span 80 in light mineral oil	[8]
3D microfluidic AFFD	SAP hydrogel beads containing bovine artery endothelial cells (d=114-146 μm)	<u>DP</u>: 0.5 % (w/v) PuraMatrix™ SAP solution + cell suspension in 20% (w/v) sucrose solution (1:1 volume ratio)<br><u>CP</u>: 6.8 mg mL^{-1} fine DMEM powder + 2% (w/v) Span 80 in mineral oil	[16]
Two consecutive cross-junctions	Ca-alginate beads (d=176-201 μm)	<u>DP</u>: 3 wt% sodium alginate in water<br><u>CP1</u>: 60 mPa·s corn oil<br><u>CP2</u>: 30 wt% $CaCl_2$ solution + 0.1 wt% SY-Glyster CRS-75 in corn oil	[17]
Two consecutive cross-junctions	Ca alginate beads coated Fe_3O_4 nanoparticles	<u>DP</u>: 3 wt% sodium alginate in water<br><u>CP1</u>: 60 mPa·s corn oil<br><u>CP2</u>: 2wt% Fe_3O_4 nanoparticles dispersed in 30 wt% $CaCl_2$ solution + 0.1 wt% SY-Glyster CRS-75 in corn oil	[17]
Microfluidic manifold with a single pump	Ca alginate beads containing embryonic carcinoma cells (d≈130μm)	<u>DP</u>: 2 %(w/v) sodium alginate in DMEM containing cell suspension<br><u>CP</u>: calcified oleic acid obtained by removing alcohol from alcohol-oleic acid-$CaCl_2$ mixture	[18]
MFFD with a cell-scattering microencapsulation device	Ca alginate beads containing embryonic carcinoma cells (d≈90μm)	<u>DP1</u>: 3.6% (w/v) sodium alginate in α–MEM<br><u>DP2</u>: cell suspension in 1.8% (w/v) alginate solution<br><u>CP</u>: Calcified oleic acid	[19]
Microfluidic junction combined with cross junction	Ca-alginate/Matrigel composite beads containing human cervical carcinoma cells (d=250-270 μm)	<u>DP1</u>: 50% (v/v) Matrigel + 10^7 mL^{-1} human cervical carcinoma cells in DMEM<br><u>DP2</u>: 2 wt% sodium alginate in DMEM<br><u>CP</u>: 5 wt% Span 80 in mineral oil<br><u>Gelation bath</u>: 4 wt% $CaCl_2$ in DMEM	[20]

X-junction 3D flow focusing chip	Polydisperse Ca-alginate beads	DP: 2 wt% sodium alginate in water<br>CP: 1 wt% Span 80 in calcified oleic acid	[18]

Table S3. Microgels manufactured by in-drop mixing in microfluidic devices.

Microfluidic device	Microgels	Emulsion formulation	Ref.
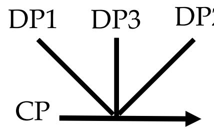	Calcium alginate beads containing yeast cells (d=60-95μm)	DP1: 1 wt% sodium alginate in water<br>DP2: 30 mM $CaCl_2$ in water<br>DP3: yeast cells in a buffer solution<br>CP: 1 wt% Span 80 in hexadecane	[21]
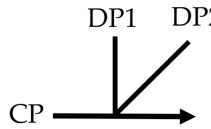	Calcium alginate beads	DP1: 172 mM GDL in water<br>DP2: 0.9 wt% sodium alginate + 22 mM $CaCO_3$ particles (dp<5 μm) in water<br>CP: Sunflower oil	[22]
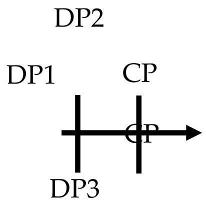	Calcium alginate beads (d=50-300μm)	DP1: Deionised water<br>DP2: 2 wt% sodium alginate in water<br>DP3: 2 wt% $CaCl_2$ in water<br>CP: Octyl alcohol	[23]
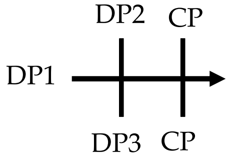	Calcium alginate beads	DP1: Deionised water<br>DP2: 0.2-2.0 wt% sodium alginate in water<br>DP3: 0.2-2.0 wt% $CaCl_2$ in water<br>CP: 3wt% fluorosurfactant in Fluorinert™ FC-40 fluoro-carbon oil	[24]
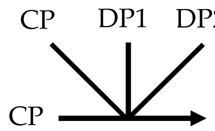	Calcium alginate beads containing LCC6/Her-2 breast tumor cells	DP1: 2wt% sodium alginate + 1×10^7 of cells mL^{-1} + 50 mM tris-HCl in DMEM<br>DP2: 40 mM $CaCl_2$ in 50 mM tris-HCl at pH 7.8<br>CP: 2% Span 80 in n-hexadecane	[25]

Table S4. Microgels manufactured by droplet merging in microfluidic devices. GOx: glucose oxidase; HEPES: (4-(2-hydroxyethyl)-1-piperazineethanesulfonic acid); HepG2, human liver cancer cell line; HRP: horseradish peroxidase; MEL: mouse erythroleukemia； MFFD: microfluidic flow focusing device.

Microfluidic device	Microgels	Emulsion formulation	Ref.
Microfluidic cross junction	PuraMatrix™ beads containing HepG2 cells (d=40-100 μm)	DP1: 2% Puramatrix in water<br>DP2: HepG2 cells in cell media<br>CP: 0.5 wt% Span 80 in mineral oil	[26]

Microfluidic cross junction	Calcium alginate beads containing glucose oxidase assay (d=40-100 μm)	DP1: 2 (w/v)% alginic acid + 2 U/mL GOx + 0.2 U/mL HRP + 100 μM Amplex Red in water<br>DP2: 0.5 mM $CaCl_2$ in water<br>CP: 0.5 wt% Span 80 in mineral oil	[26]
MFFD with fusion chambers	Disk-like, plug-like and spherical calcium alginate beads (d=20-50 μm)	DP1: sodium alginate in water<br>DP2: $CaCl_2$ in water<br>CP: Soybean oil	[27]
Microfluidic Ψ-junction	Calcium alginate beads and fibres containing living cells (d=50-200 μm)	DP1: 0.5-1% sodium alginate + 3T3 and L929 fibroblast cells in water<br>DP2: 0.13 M $CaCl_2$ in water<br>CP: Soybean oil	[28]
MFFD with fusion chamber	Ca-alginate/magnetic Janus particles (d=48-62 μm)	DP1: 2.67 wt% sodium alginate + $2.13{\times}10^{11}$ mL^{-1} magnetic NPs in water<br>DP2: 2 wt% sodium alginate in water<br>DP3: 1 wt% $CaCl_2$ in water<br>CP: Soybean oil	[29]
Straight through silicon micronozzles	Calcium alginate beads containing living cells (d=50-200 μm)	DP1: 1.5% sodium alginate + $1{\times}10^7$ cells/ml in HEPES buffer solution<br>DP2: 0.1M $CaCl_2$ + 0.14 M NaCl in water<br>CP: Soybean oil	[30]
Microfluidic Y-junction with a nozzle	Calcium alginate beads containing MEL cells (d=104-167 μm)	DP1: 1.5 wt% sodium alginate + $1{\times}10^5$ mL^{-1} cells in HEPES buffer solution<br>DP2: 0.1M $CaCl_2$ solution<br>CP: Edible oil	[31]
MFFD with two expansion chambers and a serpentine channel	Calcium alginate beads with Fe_3O_4 nanoparticles containing cancer HCT116 cells (d=21 μm)	DP1: 2 wt% sodium alginate + Fe_3O_4 nanoparticles (250 nm) + $1{\times}10^5$ mL^{-1} HCT116 cells in water<br>DP2: 1 wt% $CaCl_2$ in water<br>CP: soybean oil stained with oil-soluble dye Sudan III	[32]
X- and T-junction droplet chip	Multi-compartment network of Ca alginate	DP1: 2wt% sodium alginate in water<br>CP1: 1 wt% Span 80 in oleic acid<br>CP2: 1 wt% $CaCl_2$	[33]

Table S5. Microgels manufactured by enzymatic crosslinking in microfluidic devices. CF-KRH: calcium-free Krebs Ringer Hepes buffered solution; CRFK: Crandall-Reese feline kidney cells; FBS: fetal bovine serum; hMSCs: human mesenchymal stem cells; HRP: Horseradish Peroxidase; PBS: Phosphate-buffered saline; MIN6-B1: mouse insulinoma cell.

Microfluidic device	Microgels	Emulsion formulation	Ref.
Microfluidic co-flow device	Alginate-tyramine beads containing cells (d=80-280 μm)	DP: 1.5 %(w/v) Alginate-tyramine + 1.6 U/mL HRP + 1.5×10^{7} mL^{-1} CRFK cells in CF-KRH. CP: 0.82 mM H_2O_2 + 3 wt% lecithin in liquid paraffin	[34]
Microfluidic glass capillary device	Dextran-tyramine beads containing hMSCs (d=80-300 μm)	DP1: 2.5, 5, 10 % (w/v) Dextran-tyramine + 250 U/mL HRP + 2×10^{7} mL^{-1} hMSCs cells in DMEM/FBS mixture CP: 1% (w/v) Span 80 in n-hexadecane H_2O_2 bath: 10%, 20%, 30% H_2O_2 solution	[35]
Microfluidic flow-focusing device	Dextran-Tyramine conjugates beads containing MIN6-B1 cells	DP: 5% (w/v) Dextran-tyramine conjugates + 22 U/mL HRP + 83000 U/mL catalase + 7.5×10^{7} cells/mL MIN6-B1 cells in PBS buffer CP: 1% (w/v) Span 80 in hexadecane+30% (w/v) H_2O_2	[36]
Flow-focusing device with 3 converging inlet channels	Dex-TA beads containing MSCs	DP1: 10% (w/v) Dextran-tyramine + 1×10^{7} cells/mL MSCs in PBS buffer DP2: 44 U/ml HRP + 8% (w/v) iodixanol in PBS buffer DP3: 5×10^{-6} M H_2O_2 CP: 2% biocompatible surfactant in fluorocarbon oil	[37]
Microfluidic flow-focusing device	Dex-HA-TA beads containing MSCs	DP1: 5-10% (w/v) Dex-tyramine + 5% (w/v) hyaluronic acid-tyramine + 1×10^{7} cells/mL MSCs in PBS buffer DP2: 44 U/ml HRP + 8% (w/v) iodixanol in PBS buffer DP3: 5×10^{-6} M H_2O_2 CP: 2% biocompatible surfactant in fluorocarbon oil	[37]

Table S6. Microgels manufactured by photo-crosslinking n in microfluidic devices. ABIL® EM 90: Cetyl PEG/PPG-10/1 dimethicone; Dex-HEMA: Dextran-hydroxyethyl methacrylate; DMMI: dimethylmaleimide; GelMA: Gelatin-methacryloyl; Igepal: octylyphen oxypolyethoxyethanol; Irgacure 2959: 2-Hydroxy-4′-(2-hydroxyethoxy)-2-methylpropiophenone; PEGDA: Poly(ethylene glycol diacrylate); PEGMA: Poly(ethylene glycol methyl ether acrylate); NIPAAm: N-isopropylacrylamide; QD: quantum dot; TEMED: tetramethyl ethylenediamine; TXS: thioxanthone-2,7-disulfonate.

Microfluidic device	Microgels	Emulsion formulation	Ref.
Flow focusing PDMS device	Dex-HEMA beads (d=10 μm)	DP: 30 wt% dex-HEMA (pre-polymer) + Irgacure 2959 in water<br>CP: 4 vol% ABIL® EM 90 in mineral oil	[38]
Flow-focusing device	GelMA beads (d=35-150 μm)	DP: 8 wt% GelMA + 0.2 wt% Irgacure 2959 in PBS at pH 7.4<br>CP: 20 wt% Span 80 in mineral oil	[39]
Microfluidic flow-focusing device	pNIPPAm beads (d=60-150μm)	DP:50 g/L P(NIPAAm-DMMI) + 0.5 mmol/L TXS<br>CP: 2 wt% ABIL® EM 90 in paraffin oil	[40]
MFFD with wavy downstream channel	QD-encoded PEGDA beads (d=10 μm, 18 μm, 39 μm)	DP: 5 mg/mL Irgacure 2959 + QDs in PEGDA<br>CP: 4wt% ABIL® EM 90 in hexadecane	[41]
MFFD with wavy downstream channel	PEGDA beads	DP: 0.1 mg/mL Rhodamine B + 1% (w/v) Irgacure 2959 + 25%, 50%, or 100% (w/v) PEGDA in water<br>CP: 1% (w/v) TEMED + ABIL® EM 180 in mineral oil	[42]
MFFD with wavy downstream channel	PEGMA or PEGDA beads (d=40-200 μm)	DP: 20, 40 or 60 wt% PEGMA or PEGDA + 3 wt% Irgacure 2959 + 1wt% Igepal + 10 mM rhodamine B<br>CP: Mineral oil	[43]

Table S7. Core-shell microgels manufactured by external gelation in microfluidic devices. PGPR 90: Polyglycerol polyricinoleate; Tween 20: Polysorbate 20.

Microfluidic device	Microgels	Emulsion formulation	Ref.
Three-phase glass capillary device	Gellan gum shell with a sunflower oil core (d=95-260 μm)	O_1: Sunflower oil labelled with an orange dye<br>W: 0.5 wt% gellan gum + 2 wt% Tween 20 in water<br>O_2: 1 wt% calcium acetate + 5 wt% PGPR 90 in sunflower oil	[44]
Co-flow glass capillary device	Alginate shell with a soybean oil core (d=250-255 μm)	O_1: 8%(w/v) PGPR 90 in soybean oil<br>W_1: 2% (w/v) sodium alginate in water<br>O_2: 8 % (w/v) PGPR 90 in soybean oil<br>W_2: 10 % (w/v) $CaCl_2$ in water	[45]

Table S8. Core-shell microgels manufactured by internal gelation of charged polymers in microfluidic devices: DMEM: Dulbecco's Modified Eagle Medium; EDTA: Ethylenediaminetetraacetic acid; HepG2: human hepatocellular carcinoma cells; HFE-7500; fluorinated oil; LR300: Lumogen® F Red 300; PAG: photo-acid generator (diphenyl iodonium nitrate); PFPE / PEG: perfluoropolyether / poly(ethylene glycol); PGPR 90: Polyglycerol polyricinoleate.

Microfluidic device	Microgels	Emulsion formulation	Ref.
Co-flow glass capillary device	Alginate shell with a single or multiple/distinct oil cores	$\underline{O_1}$: 2% (v/w) PGPR 90 + 33% (v/v) thyme or lavender essential oil in a 1:1 (v/v) soybean oil/ benzyl benzoate mixture<br>W: 0.5-2.5 % (w/v) sodium alginate + 0.5% (w/v) Pluronic F-127+ 0.1 mM Ca-EDTA<br>$\underline{O_2}$: 5% (w/v) PGPR 90 + in soybean oil<br>Gelation bath: 5% (w/v) acetic acid + 5% (w/v) PGPR 90 in soybean oil	[46]
Co-flow glass capillary device	Alginate shell with a soybean oil core	$\underline{O_1}$: LR300 in a 1:1 (v/v) soybean oil/ benzyl benzoate mixture<br>W: 2 wt% sodium alginate + 0.15 (w/v) $CaCO_3$ nanoparticles + 30 mM PAG +1 wt% Pluronic F127 in water<br>$\underline{O_2}$: 8% (w/v) PGPR 90 in soybean oil	[47]
Microfluidic flow-focusing device	Alginate shell with aqueous core containing HepG2 cells	$\underline{W_1}$: 1×10^8 HepG2 cells/mL in DMEM<br>$\underline{W_2}$: 100 mM Ca-EDTA + 1.7% (w/v) alginate in water<br>$\underline{O_1}$: 1% (w/v) PFPE/PEG in HFE-7500<br>$\underline{O_2}$: 1% (w/v) PFPE/PEG +0.15% (w/v) acetic acid in HFE-7500	[48]

Table S9. Core-shell microgels manufactured by photo-crosslinking in microfluidic devices. ABIL® EM 90: Cetyl PEG/PPG-10/1 dimethicone; DEX-GPE: glycidyl propargyl ether derivatized dextran: DEX-SH: thiol derivatized dextran; GelMA: Gelatin-methacryloyl; MC: Methyl cellulose; PNIPAAm: poly(N-isopropylacrylamide); PAAm: Poly(acrylamide); PEG: Polyethylene glycol; PGPR: Polyglycerol polyricinoleate; TXS: thioxanthone-2,7-disulfonate.

Microfluidic device	Microgels	Emulsion formulation	Ref.
3D PDMS MFFD with two cross junctions	Dextran shell with a PEG core (d=130-230 μm)	Inner: 10 wt% PEG + 3 mg/mL Irgacure 2959 in water<br>Middle: 5 wt% DEX-GPE + 5 wt% DEX-SH + 3 mg/mL Irgacure 2959 in water<br>Outer: 40 wt% PEG + 5 mg/mL Irgacure 2959 in water	[49]
MFFD with two cross junctions	GelMA shell with a methyl cellulose core (d=279-367 μm)	Inner: 1 wt% MC in water<br>Middle: 8 wt% GelMA + 0.5 wt% Irgacure 2959 in water<br>Outer: 2 wt% Span 80 in mineral oil	[50]
MFFD with two cross junctions	p(NIPAAm) shell with a PAAm core (d≈120 μm)	Inner: pNIPAAm beads dispersed in water<br>Middle: 35g/L P(NIPAAm-DMMI) + 15 g/L green-tagged P(NIPAAm-DMMI) + 0.5 mM TXS in water<br>Outer: 2wt% ABIL® EM 90 in paraffin oil	[51]

References

1. Zhang, H.; Tumarkin, E.; Sullan, R. M. A.; Walker, G. C.; Kumacheva, E. Exploring Microfluidic Routes to Microgels of Biological Polymers. *Macromol. Rapid Commun.* **2007**, *28*, 527–538, doi:10.1002/marc.200600776.
2. Capretto, L.; Mazzitelli, S.; Balestra, C.; Tosi, A.; Nastruzzi, C. Effect of the Gelation Process on the Production of Alginate Microbeads by Microfluidic Chip Technology. *Lab on a Chip* **2008**, *8*, 617–621, doi:10.1039/b714876c.
3. Tan, W. H.; Takeuchi, S. Monodisperse Alginate Hydrogel Microbeads for Cell Encapsulation. *Adv. Mater.* **2007**, *19*, 2696–2701, doi:10.1002/adma.200700433.
4. Utech, S.; Prodanovic, R.; Mao, A. S.; Ostafe, R.; Mooney, D. J.; Weitz, D. A. Microfluidic Generation of Monodisperse, Structurally Homogeneous Alginate Microgels for Cell Encapsulation and 3D Cell Culture. *Adv. Healthc. Mater.* **2015**, *4*, 1628–1633, doi:10.1002/adhm.201500021.
5. Liu, H.; Li, G.; Sun, X.; He, Y.; Sun, S.; Ma, H. Microfluidic Generation of Uniform Quantum Dot-Encoded Microbeads by Gelation of Alginate. *RSC Adv.* **2015**, *5*, 62706–62712, doi:10.1039/c5ra10688e.
6. Morimoto, Y.; Tan, W. H.; Tsuda, Y.; Takeuchi, S. Monodisperse Semi-Permeable Microcapsules for Continuous Observation of Cells. *Lab on a Chip* **2009**, *9*, 2217–2223, doi:10.1039/b900035f.
7. Marquis, M.; Davy, J.; Fang, A.; Renard, D. Microfluidics-Assisted Diffusion Self-Assembly: Toward the Control of the Shape and Size of Pectin Hydrogel Microparticles. *Biomacromolecules* **2014**, *15*, 1568–1578, doi:10.1021/bm401596m.
8. Carreras, M. P.; Wang, S. A Multifunctional Microfluidic Platform for Generation, Trapping and Release of Droplets in a Double Laminar Flow. *J. Biotechnol.* **2017**, *251* , 106–111, doi:10.1016/j.jbiotec.2017.04.030.
9. Enck, K. E. E.; Ajan, S. H. P. R. R.; Leman, J. U. A.; Astagno, S. I. C.; Ong, E. M. L.; Halil, F. A. K.; All, A. D. A. M. R. H.; Para, E. M. C. O. Design of an Adhesive Film-Based Microfluidic Device for Alginate Hydrogel-Based Cell Encapsulation. **2020**, *48*, 1103–1111, doi:10.1007/s10439-020-02453-9.
10. Workman, V. L.; Dunnett, S. B.; Kille, P.; Palmer, D. D. On-Chip Alginate Microencapsulation of Functional Cells. *Macromol. Rapid Commun.* **2008**, *29*, 165–170, doi:10.1002/marc.200700641.
11. Marquis, M.; Renard, D.; Cathala, B. Microfluidic Generation and Selective Degradation of Biopolymer-Based Janus Microbeads. *Biomacromolecules* **2012**, *13*, 1197–1203, doi:10.1021/bm300159u.
12. Zhang, H.; Tumarkin, E.; Peerani, R.; Nie, Z.; Sullan, R. M. A.; Walker, G. C.; Kumacheva, E. Microfluidic Production of Biopolymer Microcapsules with Controlled Morphology. *J. Am. Chem. Soc.* **2006**, *128*, 12205–12210, doi:10.1021/ja0635682.
13. Ogończyk, D.; Siek, M.; Garstecki, P. Microfluidic Formulation of Pectin Microbeads for Encapsulation and Controlled Release of Nanoparticles. *Biomicrofluidics* **2011**, *5*, 1–12, doi:10.1063/1.3569944.
14. Kim, C.; Park, K. S.; Kim, J.; Jeong, S. G.; Lee, C. S. Microfluidic Synthesis of Monodisperse Pectin Hydrogel Microspheres Based on in Situ Gelation and Settling Collection. *J. Chem. Technol. Biotechnol.* **2017**, *92*, 201–209, doi:10.1002/jctb.4991.
15. Song, Y.; Lee, C. S. In Situ Gelation of Monodisperse Alginate Hydrogel in Microfluidic Channel Based on Mass Transfer of Calcium Ions. *Korean Chem. Eng. Res.* **2014**, *52*, 632–637, doi:10.9713/kcer.2014.52.5.632.
16. Tsuda, Y.; Morimoto, Y.; Takeuchi, S. Monodisperse Cell-Encapsulating Peptide Microgel Beads for 3D Cell Culture. *Langmuir* **2010**, *26*, 2645–2649, doi:10.1021/la902827y.
17. Liu, Y.; Tottori, N.; Nisisako, T. Microfluidic Synthesis of Highly Spherical Calcium Alginate Hydrogels Based on External Gelation Using an Emulsion Reactant. *Sensors Actuators, B Chem.* **2019**, *283*, 802–809, doi:10.1016/j.snb.2018.12.101.
18. Kim, C.; Park, J.; Kang, J. Y. A Microfluidic Manifold with a Single Pump System to Generate Highly Mono-Disperse Alginate Beads for Cell Encapsulation. *Biomicrofluidics* **2014**, *8*, 1–10, doi:10.1063/1.4902943.
19. Kim, C. Droplet-Based Microfluidics for Making Uniform-Sized Cellular Spheroids in Alginate Beads with the Regulation of Encapsulated Cell Number. *Biochip J.* **2015**, *9*, 105–113, doi:10.1007/s13206-015-9203-6.
20. Wang, Y.; Wang, J. Mixed Hydrogel Bead-Based Tumor Spheroid Formation and Anticancer Drug Testing. *Analyst* **2014**, *139*, 2449–2458, doi:10.1039/c4an00015c.
21. Choi, C. H.; Jung, J. H.; Rhee, Y. W.; Kim, D. P.; Shim, S. E.; Lee, C. S. Generation of Monodisperse Alginate Microbeads and in Situ Encapsulation of Cell in Microfluidic Device. *Biomed. Microdevices* **2007**, *9*, 855–862, doi:10.1007/s10544-007-9098-7.
22. Amici, E.; Tetradis-Meris, G.; de Torres, C. P.; Jousse, F. Alginate Gelation in Microfluidic Channels. *Food Hydrocoll.* **2008**, *22*, 97–104, doi:10.1016/j.foodhyd.2007.01.022.
23. Xu, J. H.; Li, S. W.; Tan, J.; Luo, G. S. Controllable Preparation of Monodispersed Calcium Alginate Microbeads in a Novel Microfluidic System. *Chem. Eng. Technol.* **2008**, *31*, 1223–1226, doi:10.1002/ceat.200800027.
24. Mazutis, L.; Vasiliauskas, R.; Weitz, D. A. Microfluidic Production of Alginate Hydrogel Particles for Antibody Encapsulation and Release. *Macromol. Biosci.* **2015**, *15*, 1641–1646, doi:10.1002/mabi.201500226.
25. Yu, L.; Chen, M. C. W.; Cheung, K. C. Droplet-Based Microfluidic System for Multicellular Tumor Spheroid Formation and Anticancer Drug Testing. *Lab on a Chip* **2010**, *10*, 2424–2432, doi:10.1039/c004590j.
26. Um, E.; Lee, D. S.; Pyo, H. B.; Park, J. K. Continuous Generation of Hydrogel Beads and Encapsulation of Biological Materials Using a Microfluidic Droplet-Merging Channel. *Microfluid. Nanofluidics* **2008**, *5*, 541–549, doi:10.1007/s10404-008-0268-6.
27. Liu, K.; Ding, H. J.; Liu, J.; Chen, Y.; Zhao, X. Z. Shape-Controlled Production of Biodegradable Calcium Alginate Gel Microparticles Using a Novel Microfluidic Device. *Langmuir* **2006**, *22*, 9453–9457, doi:10.1021/la061729+.
28. Hong, J. S.; Shin, S. J.; Lee, S. H.; Wong, E.; Cooper-White, J. Spherical and Cylindrical Microencapsulation of Living Cells Using Microfluidic Devices. *Korea Aust. Rheol. J.* **2007**, *19*, 157–164.

29. Zhao, L. B.; Pan, L.; Zhang, K.; Guo, S. S.; Liu, W.; Wang, Y.; Chen, Y.; Zhao, X. Z.; Chan, H. L. W. Generation of Janus Alginate Hydrogel Particles with Magnetic Anisotropy for Cell Encapsulation. *Lab on a Chip* **2009**, *9*, 2981–2986, doi:10.1039/b907478c.
30. Sugiura, S.; Oda, T.; Izumida, Y.; Aoyagi, Y.; Satake, M.; Ochiai, A.; Ohkohchi, N.; Nakajima, M. Size Control of Calcium Alginate Beads Containing Living Cells Using Micro-Nozzle Array. *Biomaterials* **2005**, *26*, 3327–3331, doi:10.1016/j.biomaterials.2004.08.029.
31. Shintaku, H.; Kuwabara, T.; Kawano, S.; Suzuki, T.; Kanno, I.; Kotera, H. Micro Cell Encapsulation and Its Hydrogel-Beads Production Using Microfluidic Device. *Microsyst. Technol.* **2007**, *13*, 951–958, doi:10.1007/s00542-006-0291-z.
32. Liu, K.; Deng, Y.; Zhang, N.; Li, S.; Ding, H.; Guo, F.; Liu, W.; Guo, S.; Zhao, X. Z. Generation of Disk-like Hydrogel Beads for Cell Encapsulation and Manipulation Using a Droplet-Based Microfluidic Device. *Microfluid. Nanofluidics* **2012**, *13*, 761–767, doi:10.1007/s10404-012-0998-3.
33. Damiati, S. In Situ Microfluidic Preparation and Solidification of Alginate Microgels. *Macromolecular Research* **2020**, *28*, 1046–1053, doi:10.1007/s13233-020-8142-9.
34. Sakai, S.; Hashimoto, I.; Ogushi, Y.; Kawakami, K. Peroxidase-Catalyzed Cell Encapsulation in Subsieve-Size Capsules of Alginate with Phenol Moieties in Water-Immiscible Fluid Dissolving H2O2. *Biomacromolecules* **2007**, *8*, 2622–2626, doi: 10.1021/bm070300+.
35. Henke, S.; Leijten, J.; Kemna, E.; Neubauer, M.; Fery, A.; van den Berg, A.; van Apeldoorn, A.; Karperien, M. Enzymatic Crosslinking of Polymer Conjugates Is Superior over Ionic or UV Crosslinking for the On-Chip Production of Cell-Laden Microgels. *Macromol. Biosci.* **2016**, *16*, 1524–1532, doi:10.1002/mabi.201600174.
36. Kamperman, T.; Henke, S.; Zoetebier, B.; Ruiterkamp, N.; Wang, R.; Pouran, B.; Weinans, H.; Karperien, M.; Leijten, J. Nanoemulsion-Induced Enzymatic Crosslinking of Tyramine-Functionalized Polymer Droplets. *R. Soc. Chem.* **2017**, *5*, 4835–4844, doi:10.1039/c7tb00686a.
37. Kamperman, T.; Henke, S.; Visser, C. W.; Karperien, M. Centering Single Cells in Microgels via Delayed Crosslinking Supports Long-Term 3D Culture by Preventing Cell Escape. *Small* **2017**, *13*, 1603711, doi:10.1002/smll.201603711.
38. Geest, B. G. De; Urbanski, J. P.; Thorsen, T.; Demeester, J.; Smedt, S. C. De. Synthesis of Monodisperse Biodegradable Microgels in Microfluidic Devices. *Langmuir* **2005**, *21*, 10275–10279, doi:10.1021/la051527y.
39. Cha, C.; Oh, J.; Kim, K.; Qiu, Y.; Joh, M.; Shin, S. R.; Wang, X.; Camci-Unal, G.; Wan, K. T.; Liao, R.; Khademhosseini, A. Microfluidics-Assisted Fabrication of Gelatin-Silica Core-Shell Microgels for Injectable Tissue Constructs. *Biomacromolecules* **2014**, *15*, 283–290, doi:10.1021/bm401533y.
40. Seiffert, S.; Weitz, D. A. Controlled Fabrication of Polymer Microgels by Polymer-Analogous Gelation in Droplet Microfluidics. *Soft Matter* **2010**, *6*, 3184–3190, doi:10.1039/c0sm00071j.
41. Liu, H.; Qian, X.; Wu, Z.; Yang, R.; Sun, S.; Ma, H. Microfluidic Synthesis of QD-Encoded PEGDA Microspheres for Suspension Assay. *J. Mater. Chem. B* **2016**, *4*, 482–488, doi:10.1039/c5tb02209f.
42. Nozdriukhin, D. V; Filatov, N. A.; Evstrapov, A. A.; Bukatin, A. S. Formation of Polyacrylamide and PEGDA Hydrogel Particles in a Microfluidic Flow Focusing Droplet Generator. **2018**, *63*, 1371–1376, doi:10.1134/S1063784218090141.
43. Dang, T.; Ho, Y.; Gon, H.; Man, G. Preparation of Monodisperse PEG Hydrogel Microparticles Using a Microfluidic Flow-Focusing Device. *J. Ind. Eng. Chem.* **2012**, *18*, 1308–1313, doi:10.1016/j.jiec.2012.01.028.
44. Michelon, M.; Leopércio, B. C.; Carvalho, M. S. Microfluidic Production of Aqueous Suspensions of Gellan-Based Microcapsules Containing Hydrophobic Compounds. *Chem. Eng. Sci.* **2020**, *211*, 115314, doi:10.1016/j.ces.2019.115314.
45. Ren, P. W.; Ju, X. J.; Xie, R.; Chu, L. Y. Monodisperse Alginate Microcapsules with Oil Core Generated from a Microfluidic Device. *J. Colloid Interface Sci.* **2010**, *343*, 392–395, doi:10.1016/j.jcis.2009.11.007.
46. Mou, C. L.; Deng, Q. Z.; Hu, J. X.; Wang, L. Y.; Deng, H. B.; Xiao, G.; Zhan, Y. Controllable Preparation of Monodisperse Alginate Microcapsules with Oil Cores. *J. Colloid Interface Sci.* **2020**, *569*, 307–319, doi:10.1016/j.jcis.2020.02.095.
47. Liu, L.; Wu, F.; Ju, X.; Xie, R.; Wang, W.; Hui, C.; Chu, L. Preparation of Monodisperse Calcium Alginate Microcapsules via Internal Gelation in Microfluidic-Generated Double Emulsions. *J. Colloid Interface Sci.* **2013**, *404*, 85–90, doi:10.1016/j.jcis.2013.04.044.
48. Chen, Q.; Utech, S.; Chen, D.; Prodanovic, R.; Lin, J. M.; Weitz, D. A. Controlled Assembly of Heterotypic Cells in a Core-Shell Scaffold: Organ in a Droplet. *Lab on a Chip* **2016**, *16*, 1346–1349, doi:10.1039/c6lc00231e.
49. Mytnyk, S.; Ziemecka, I.; Olive, A. G. L.; Van der Meer, J. W. M.; Totlani, K. A.; Oldenhof, S.; Kreutzer, M. T.; Van Steijn, V.; Van Esch, J. H. Microcapsules with a Permeable Hydrogel Shell and an Aqueous Core Continuously Produced in a 3D Microdevice by All-Aqueous Microfluidics. *RSC Adv.* **2017**, *7*, 11331–11337, doi:10.1039/c7ra00452d.
50. Wang, H.; Liu, H.; Liu, H.; Su, W.; Chen, W.; Qin, J. One-Step Generation of Core – Shell Gelatin Methacrylate (GelMA) Microgels Using a Droplet Microfluidic System. *Advanced Materials Technologies* **2019**, *4*, 1800632, doi:10.1002/admt.201800632.
51. Seiffert, S.; Thiele, J.; Abate, A. R.; Weitz, D. A. Smart Microgel Capsules from Macromolecular Precursors. *Journal of the American Chemical Society* **2010**, *132*, 6606-6609, doi:10.1021/ja102156h.
